# Generating value with mental health apps

**DOI:** 10.1192/bjo.2019.98

**Published:** 2020-02-05

**Authors:** Adam C. Powell, John B. Torous, Joseph Firth, Kenneth R. Kaufman

**Affiliations:** President, Payer+Provider Syndicate, USA; Director, Digital Psychiatry Division, Department of Psychiatry, Beth Israel Deaconess Medical Center, Harvard Medical School, USA; Senior Research Fellow, NICM Health Research Institute, Western Sydney University, Australia; and Honorary Research Fellow, Division of Psychology and Mental Health, Faculty of Biology Medicine and Health, University of Manchester, UK; Professor of Psychiatry, Neurology, and Anaesthesiology, Rutgers Robert Wood Johnson Medical School, USA; and Visiting Professor of Psychological Medicine, Institute of Psychiatry, Psychology & Neuroscience, King's College London, UK

**Keywords:** Health insurance, mHealth, mobile health, smartphones, managed care, value, apps, digital

## Abstract

**Background:**

Although apps are increasingly being used to support the diagnosis, treatment and management of mental illness, there is no single means through which costs associated with mental apps are being reimbursed. Furthermore, different apps are amenable to different means of reimbursement as not all apps generate value in the same way.

**Aims:**

To provide insights into how apps are currently generating value and being reimbursed across the world, with a particular focus on the situation in the USA.

**Method:**

An international team performed secondary research on how apps are being used and on common pathways to remuneration.

**Results:**

The uses of apps today and in the future are reviewed, the nature of the value delivered by apps is summarised and an overview of app reimbursement in the USA and other countries is provided. Recommendations regarding how payments might be made for apps in the future are discussed.

**Conclusions:**

Currently, apps are being reimbursed through channels with other original purposes. There may be a need to develop an app-specific channel for reimbursement which is analogous to the channels used for devices, drugs and laboratory tests.

The use of apps to support mental healthcare is evolving. As apps play an expanding role in care, their complexity is in some cases simultaneously increasing. To cover the costs associated with more complex apps being used for a diverse set of purposes, app developers have sought multiple pathways to reimbursement. International differences in the financing of healthcare have likewise led to different approaches being pursued in different countries. The aim of this article is to provide insights into how apps are currently generating value and being reimbursed across the world, with a particular focus on the situation in the USA.

## Method

An international team performed secondary research on how apps are being used in the context of mental health and on common pathways to remuneration. The team featured members with experience working in Australia, the UK and the US. Team members collaboratively developed conclusions based on their findings.

## Results

### Use of apps today

Today, apps are being used to support the diagnosis, treatment and management of mental illness in multiple ways. Apps are used in the context of preventive, acute and even emergency care. There are over 250 000 health apps available according to industry reports.^1^ The USA iOS App Store contains over 300 apps to address anxiety disorder alone.^2^ Nonetheless, there is no single means through which costs associated with mental health apps are being reimbursed.^3^

Although much remains unknown about exactly who is using mental health apps, it is clear that many are interested and likely trying them.^4^ In 2015, a national survey noted that mental health apps were in the top three categories of health apps downloaded.^5^ Apps have the potential to increase access to care for people who might not otherwise seek professional care, as patients may use apps independently or be encouraged by apps to seek in-person help via app-based screening and diagnosis. One of the earliest examples is a 2014 study that offered depression screening to over 8000 people across 66 countries and encouraged those with elevated nine-item Patient Health Questionnaire scores to seek local help.^6^ Since then, a plethora of screening apps have emerged.

Apps can also benefit those already in care. For example, people who have sought professional care are using apps both to interact with their healthcare provider via an additional channel, and to assess and treat their conditions on a standalone basis. This hybrid approach of integrating apps to augment care has been implemented at community clinics^7^ and the Veterans Administration.^8^ Furthermore, healthcare providers are in some instances using apps to support administrative aspects regarding the delivery of care, such as care management and case management.^9^ Thus, mental health apps exist along a spectrum – some are used as a complete substitute for in-person care, and others are used behind the scenes to support in-person processes. Although many apps today are not regulated in the USA either because they claim to be wellness tools (rather than medical devices) or are in categories subject to regulatory discretion, several are pursuing formal US Food and Drug Administration approval.^10^

Apps are as diverse in their quality as they are in their purposes. Reviews of apps on the iTunes and Google Play commercial marketplaces suggest that most mental health apps do not follow evidence-based guidelines^11^ and many actually pose privacy risks to patients using them.^12^ Nonetheless, there also are evidence-based apps, such as the IntelliCare apps developed at Northwestern University,^13^ which stand in contrast to most of the apps available.

### Use of apps in the future

The three locations in which apps are used – outside of a clinical setting, within a mental health clinical setting, and within other clinical settings (for example the emergency department, the primary care physician's office) – are not likely to change in years to come. However, the extent, level of integration and level of ubiquity of app use is likely to increase over time as patient access to smartphones only increases and app offerings become more advanced. In the end state, there will likely not be a distinction made as to whether apps are employed in the care-delivery process or not, as they will be viewed as foundational infrastructure and tools, much like websites are today. Although the initial enthusiasm for apps has led to speculation that as tools, apps alone may offer effective self-help, increasing evidence suggests that a more effective use is towards augmenting and extending clinical care – not replacing it.^14^ The quality and evidence-base for apps will also likely improve. Thus, it makes sense that apps are progressively becoming part of the infrastructure for healthcare delivery as they assume their proper role. In the years that unfold, apps will be used for a variety of population health management applications, enabling healthcare providers to better manage patients across the continuum of care.

Although patients are able to use apps while at home, many apps today have little or no integration into the traditional care process, and as a result may further the fragmentation of care. In the future, the frequency with which such apps are integrated into traditional care will likely increase. Informatics standards backed by the federal government such as FHIR (Fast Healthcare Interoperability Resource) and SMART^15^ offer a clear path for this integration. By all metrics, the role of these health apps will dramatically increase as apps are created to fill a broader array of needs, apps become more tightly integrated into the clinical workflow and more evidence regarding the effectiveness of apps becomes available.

### Value delivered by apps

Value in healthcare is measured by dividing the outcomes delivered by an intervention by its cost.^16^ Apps are being employed in a diverse set of ways within mental health, and the value that they deliver to patients, healthcare providers and health plans differs according to the manner in which they are used and priced. Patients benefit from apps to the extent to which they improve the quality of their mental health – either by enabling them to access screening, diagnosis and treatment that they would otherwise not receive, or by augmenting the screening, diagnosis and treatment that they do receive. Such augmentation can involve a diverse range of tools, including apps for implementing measurement-based care, digital therapeutics that may be used at home, or care/case management software that improves continuity and coordination of care among healthcare providers. Improved mental health can in turn lead to savings from improved physical health, better earnings from enhanced current performance at work, and potentially higher future earnings because of the progressive nature of many careers. The economic burden of depression, sadness and mental illness is so large that when its costs are spread across all employees (not just those who are affected), it costs employers approximately $300 per employee per year. Thus, there is large potential savings at stake in addressing mental illness.^17^ However, realising return on this investment will not be immediate and requires a tolerance for longer-term outcomes.

Apps can also potentially reduce many of the direct and indirect costs associated with care, through enabling services to be delivered more efficiently, reducing physical health costs associated with untreated comorbid mental illness, and by reducing the need for in-person visits and the associated transportation costs. One programme found that the savings from reduced hospital admissions resulting from an app-based mental health initiative were so great that they more than offset the cost of providing patients with smartphones.^18^ Healthcare providers gain value from apps to the extent that they increase demand for mental health services, foster greater use of services (particularly, app-related services, such as brief assessments), decrease operating costs and improve patient attendance/engagement.^19^ Apps deliver value to health plans from potential savings resulting from substitution for other forms of care, as well as potential savings from the prevention of higher-acuity situations as a result of early identification and recognition.^20^

Still, the majority of health apps today have not demonstrated value and economic outcomes are sparse.^21^ A review of top-funded digital health companies examined the studies supported by these companies and found that those in mental health and depression have examined feasibility, but they have not yet examined clinical effectiveness.^22^ There have been multiple attempts in the literature to characterise the value delivered by apps when used to improve mental health, ranging from a paper that provided a framework for analyses without numerical findings^20^ to a systematic and meta-review.^23^ The meta-review considered the findings of 21 reviews, and concluded that there was a lack of evidence regarding the cost-effectiveness of digital health interventions when used by young people (adults were not explored). A broader systematic review of the literature, which considered cost-effectiveness and cost-utility studies of mobile health and telemedicine in all contexts concluded that the literature is limited and that there are frequently methodological issues with the analyses that do exist.^24^ A literature review that explored whether apps were effective in the context of mental health found a shortage of evidence, particularly for some categories of apps.^25^ In order to conclude whether apps deliver value, there needs to be first further research into the effectiveness component of the value equation. This does not mean that health apps do not generate value, but rather reflects the need for more outcomes data and real-world experiences using them in actual clinical settings,^26^ along with cost-effectiveness analyses.

Regardless of the conclusions of value-related analyses, the adoption of mental health apps will be impeded if there are not meaningful reimbursement mechanisms that are visibly available. When confidential, one-off contracts between organisations and app vendors are used to provide reimbursement, potential vendors do not have the benefit of seeing the potential financial pay-off of investing in app development, and some may forego making such investments. Given the substantial differences in which health services are financed across nations, it is unlikely that an internationally consistent reimbursement mechanism will ever cover the cost of apps. Nonetheless, if there are clear paths to reimbursement in major markets, there will be greater market support for app development activity, and there will be more visible stakeholders towards whom value-related analyses should be conducted.

### App reimbursement in the USA

The cost of apps to patients, healthcare providers and health insurers differs in accordance with how they are being used. Like pharmaceuticals, an app has a high development cost, but then next to no marginal cost to distribute an additional copy. That said, apps vary in the amount of human time input that they require: of the patient, of the healthcare provider and of other members of the care team.

**Fig. 1:**
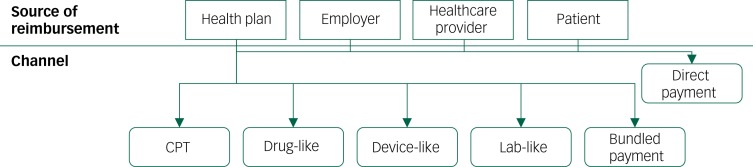
Sources of app reimbursement in the USA.^3^ Modified from reference 3. © Adam C Powell, Matthias B Bowman, Henry T Harbin. Originally published in JMIR Mental Health (http://mental.jmir.org), 06.08.2019. (An open-access article distributed under the terms of the Creative Commons Attribution License – https://creativecommons.org/licenses/by/4.0/.)

As a result of this high degree of variation, there are multiple frameworks that are used to reimburse apps. As is shown in Fig. 1, apps are reimbursed by health plans via Current Procedural Terminology (CPT) codes and via channels traditionally intended for devices, drugs and laboratory tests. One-off direct payment relationships are also used by health plans, in addition to being used by employers, healthcare providers and patients that wish to cover the costs of apps and app-related services. Finally, the costs associated with apps can be covered using a portion of bundles of funds given by a health plan to a healthcare provider for a broader purpose. A portion of the money paid for a CPT code may be used to cover the cost of an app if the app helps the healthcare provider deliver the service. Likewise, capitated payments made by a health plan to a healthcare provider may be used to purchase apps. A detailed review of the specific CPT codes being used to reimburse apps in the USA today, as well as the other channels that are being used to reimburse apps, is presented in Powell *et al*, and is outside the scope of this paper.^3^

CPT, Current Procedural Terminology.

The primary means of reimbursing healthcare provider services, CPT codes, assigns reimbursement to health services based upon a combination of the physician work, malpractice expenses and practice expenses, and then adjusts for geographic differences through the Geographic Practice Cost Index.^27^ When apps are used in tight conjunction with physician services, such as situations in which they are used for administrative purposes, it is quite feasible for there to be a substantial quantity of physician work required in addition to malpractice expenses and practice expenses. These app/service combinations are good targets for CPT-based billing. However, when apps are used by patients independently, outside the physician's office, the amount of physician and staff work required is often minimal. In these cases, it is harder for their reimbursement to be shoehorned into the CPT system because they do not make substantial use of the resources that are measured when valuing CPT codes. New CPT codes are emerging that may support clinician monitoring of remote data generated by apps, although at the time of writing, it remains unknown if this can be successfully utilised.^28^ The CPT codes that are presently available, 99453, 99454 and 99457, are intended for the monitoring of physiological parameters, and are in their present form unsuitable for use in implementing measurement-based care in the context of behavioural health. Nonetheless, mental health apps are being reimbursed as if they were hardware, via the Healthcare Common Procedure Coding System (HCPCS) codes for miscellaneous durable medical equipment (E1399) and electronic medication compliance management devices (T1505).^29^ Although there are ongoing efforts and announcements around companies such as CVS Health offering health apps,^30^ the use of prescription apps reimbursed by insurers for use in clinical care remains nascent at the time of this writing.

Many apps today are direct-to-consumer and require the end user to pay out of pocket. The sudden closure of the Lantern mental health app in 2018, which the chief executive officer suggested was because of the company's decision to pursue direct-to-consumer markets, offers one striking example of the challenges of this route.^31,32^ Nonetheless, out-of-pocket payment has been a common means of financing apps as it is the simplest approach to implement and does not require the accumulation of clinical evidence.

### App reimbursement in other countries

The approaches that other countries are taking towards app reimbursement varies in accordance with their respective modes of healthcare financing. In England, where the National Health Service (NHS) provides services free at the point of use, the government is negotiating bulk buy contracts on behalf of health systems. At least one app has been purchased in bulk using such an arrangement.^33^ The NHS has additionally created a library of apps, which are either free, require payment by patients, are offered cost-free to patients with a general practitioner's prescription or referral, or are offered cost-free to patients living and working within specific geographic areas.^34,35^

The Australian government runs a website, healthdirect, intended to provide Australians with free health advice. The website includes a symptom checker that is intended to assist Australians in triaging their illnesses. An app-based version of the site is available for Android and iOS smartphones. The healthdirect website additionally recommends a number of free apps produced by the government and third parties.^36^

A toolkit prepared by the Mental Health Commission of Canada encouraged healthcare providers to consider the costs associated with apps when recommending them to patients. The toolkit suggests that as alternatives to direct patient payments for apps, health systems or health plans could pay for apps on a per-user basis, pay a fixed fee to license unlimited access for a period of time, pay an access fee or bundle the costs associated with apps into the overall cost of care billed to self-pay patients.^37^ These approaches have also been observed in the USA.

### How apps should be reimbursed in the USA in the future

As apps become more autonomous, it becomes more difficult to tie them to reimbursement methodologies based upon physician time and practice expenses. Traditionally, devices, drugs and laboratory tests have been billed using means that do not consider the degree of human involvement in care. Device billing may occur for the one-time purchase of a device (such as a cranial electrotherapy stimulation device) or on an ongoing rental basis requiring proof of utilisation (such as a continuous positive airway pressure machine). Thus, the reimbursement channels available are amendable to be used for the reimbursement of apps that are used on a standalone, prescription basis until an app-specific channel for reimbursement is developed. Currently, while there is a CPT code, 96127, that can be used for brief behavioural assessments that are conducted by patients without physician administration, there is no analogous code for autonomous treatment.^29^ Likewise, although apps can be billed as devices via the HCPCS codes E1399 and T1505, these codes were not designed to be used to cover apps.

Although some app developers have followed these approaches to reimbursement, pursuing prescription-based reimbursement is a barrier to both developers and patients. Developers need to receive approval from the US Food and Drug Administration, and healthcare providers need prescribing authority to provide patients with access to apps reimbursed through these channels. Such a system does not take advantage of the unique features and scalability of tools like apps. As mental health services are often delivered by people without prescribing authority, including social workers and psychologists (who lack prescribing authority in many states), these channels are a barrier to patient access. Professional organisations such as the American Psychiatric Association and the American Psychological Association have not yet written practice guidelines that may help define the roles and scope of app use. Reducing these barriers for apps used without healthcare provider involvement will likely play a key role in increasing development and adoption.

Nonetheless, even those capable of prescribing apps may have difficulty recommending them. Lack of guidance on standards for app quality and app-related liability may cause healthcare providers to hesitate in recommending apps.^38^ Although physicians are liable for their drug prescription decisions, liability related to apps is murkier. To clarify matters and allay professional fears, the UK-based NHS Apps Library posts the following disclaimer: ‘Any healthcare professional recommending health apps published on the NHS Apps Library is not liable for any adverse reactions or a deterioration in health experienced by users. The liability resides exclusively with the developers of the product in question and it is their responsibility to maintain compliance with the relevant regulations.^39^ No similar protections are available to American healthcare providers.

## Discussion

Apps are playing an increasing role in enabling people to receive care for mental health issues. Over time, they will become a more integral component of our healthcare infrastructure, and fewer distinctions will be drawn between care that is facilitated by an app and care that is not. However, for apps to fulfil their full potential, changes need to be made in the reimbursement mechanisms available for care delivered through apps so that it is more feasible for app developers to be compensated for the value that they are providing.

There may be a need to unlink the connection between healthcare provider compensation and time input, and to develop an app-specific channel for reimbursing apps that is analogous to the channels used for devices, drugs and laboratory tests. The channel would consist of a standardised series of HCPCS codes for app-related procedures that health plans could then adopt and use to compensate providers for app-related services. Doing this would reduce the need for the development of one-off contracts. Once these changes have been implemented, the USA will be able to more rapidly move towards a future in which apps play a more central role in mental healthcare, following the example seen in the UK in the form of the NHS Apps Library.
